# Black Seed Thymoquinone Improved Insulin Secretion, Hepatic Glycogen Storage, and Oxidative Stress in Streptozotocin-Induced Diabetic Male Wistar Rats

**DOI:** 10.1155/2018/8104165

**Published:** 2018-03-04

**Authors:** Heba M. A. Abdelrazek, Omnia E. Kilany, Muhammad A. A. Muhammad, Hend M. Tag, Aaser M. Abdelazim

**Affiliations:** ^1^Department of Physiology, Faculty of Veterinary Medicine, Suez Canal University, Ismailia, Egypt; ^2^Department of Clinical Pathology, Faculty of Veterinary Medicine, Suez Canal University, Ismailia, Egypt; ^3^Department of Pathology, Faculty of Medicine, Suez Canal University, Ismailia, Egypt; ^4^Zoology Department, Faculty of Sciences, Suez Canal University, Ismailia, Egypt; ^5^Department of Biochemistry, Faculty of Veterinary Medicine, Zagazig University, Zagazig, Egypt; ^6^Department of Basic Medical Sciences, College of Applied Medical Sciences, University of Bisha, Bisha, Saudi Arabia

## Abstract

Diabetes mellitus is one of the metabolic diseases having several complications. *Nigella sativa* oil (NSO) might have beneficial effects in the treatment of diabetic complications. Thirty-two mature male Wistar rats were equally divided into four experimental groups: control, control NSO 2 mL/kg, streptozotocin- (STZ-) induced diabetic, and diabetic (STZ-induced) treated with oral NSO 2 mg/kg for 30 days. Fasting blood glucose (FBG), insulin, and lipid profile levels were determined. Pancreatic and hepatic tissues were used for catalase and GSH. Histopathology, hepatic glycogen contents, insulin immunohistochemistry, and pancreatic islet morphometry were performed. NSO 2 mL/kg was noticed to decrease (*P* < 0.05) FBG and increase (*P* < 0.05) insulin levels in diabetic rats than in diabetic nontreated animals. Lipid profile showed significant (*P* < 0.5) improvement in diabetic rats that received NSO 2 mL/kg than in the diabetic group. Both pancreatic and hepatic catalase and GSH activities revealed a significant (*P* < 0.05) increment in the diabetic group treated with NSO than in the diabetic animals. NSO improved the histopathological picture and hepatic glycogen contents of the diabetic group as well as increased (*P* < 0.05) insulin immunoreactive parts % and mean pancreatic islet diameter. NSO exerts ameliorative and therapeutic effects on the STZ-induced diabetic male Wistar rats.

## 1. Introduction

Diabetes mellitus (DM) is considered as one of the most common chronic metabolic diseases characterized by increased blood glucose due to insulin resistance, insulin deficiency, or both [1]. Mostly, DM is associated with vascular, metabolic, neuropathic, and nephropathic disorders. Hyperglycemia and lipid profile abnormalities are the main clues for diagnosis of DM metabolic disorders [2]. Hyperglycemia is a consequence of the inability of the cells to utilize glucose and/or skeletal muscles and liver are not capable of glycogen storage [3]. Moreover, the persistent hyperglycemia in DM promoted oxidative stress through the formation or release of reactive oxygen species (ROS) and depletion of antioxidant reserve. Oxidative stress is the main cause of cardiovascular disease that results in mortalities [4, 5].

Insulin is the primary hormone which is involved in blood glucose control. Once insulin is released into the blood, it stimulates the entrance of glucose into skeletal muscles and, to a lesser extent, liver and adipose tissue via special transporters, thus controlling glucose homeostasis [6]. Insulin binding mediates much action through interaction with insulin receptors (IR) [7].

The efficient management of diabetes requires continuous control to blood sugar level to minimize the risks of diabetic complications [8]. Thus, natural and therapeutic antioxidants are one of the strategies for diabetic remedy. Although there are various categories of antidiabetic drugs, these drugs possibly possess significant side effects or are very expensive [9, 10]. In comparison to the synthetic drug, natural plants have lesser toxicity and are devoid of any side effects [11].

*Nigella sativa* (NS), a dicotyledon plant species of the family Ranunculaceae, has been used since long eras in medical and culinary fields [12]. Its plant seeds are called black cumin or black seeds [13]. Thymoquinone (2-isopropyl-5-methyl-1,4-benzoquinone) is considered the chief active principle in the NS seeds and its volatile oily extract [14]. NS has several physiological and pharmacological properties such as hepatoprotective [15, 16], immunomodulatory [17], nephroprotective [18, 19], neuroprotective [20], antimutagenic, anticancer [21], and anticonvulsant [22] effects. Moreover, it is known for its hypotensive [23] properties.

The current study aimed to investigate the potential protective actions of NSO in streptozotocin- (STZ-) induced diabetic male Wistar rats. This was done via the manipulation of blood biochemical parameters, oxidative stress, histopathology, and pancreatic insulin immunoreactive parts.

## 2. Material and Methods

### 2.1. Experimental Rats

Thirty-two adult male Wistar rats, weighing 195–205 g, were kept in metallic cages (4/cage) at Laboratory Animal House, Faculty of Veterinary Medicine, Suez Canal University, Egypt. They were kept under standard natural day-light rhythm with a temperature of 25°C (±1°C) and allowed to ad libitum diet and water supply. The animals were handled and cared according to the ethical guidelines described by Faculty of Veterinary Medicine, Suez Canal University.

### 2.2. Experiment Design

After 14 days of acclimatization, animals were divided into four equal groups:

- Control group (*n* = 8): They were normal nondiabetic rats and gavaged daily with distilled water for one month.
- *Nigella* group (*n* = 8): They were normal nondiabetic rats that were gavaged with 2 mL/kg cold-pressed NSO (Amazing Herbs Co., Turkey) containing 0.95% thymoquinone, as determined via HPLC, by gavage tube for one month.
- Diabetic group: They were STZ-induced diabetic rats and given daily oral dose of distilled water by gavage tube for one month.
- Diabetic *Nigella*-treated group: They were STZ-induced diabetic rats that were given a daily oral dose of 2 mL/kg cold-pressed NSO (Amazing Herbs Co., Turkey) containing 0.95% thymoquinone, as determined via HPLC, by gavage tube for one month.

### 2.3. Induction of Diabetes

Experimental rats from diabetic and diabetic treated with NSO groups were induced to diabetes after 16 h fasting by a sole STZ (TUKU-E Company, USA), intraperitoneal (i.p.) injection, in a dose of 45 mg/kg. STZ was freshly prepared in 0.1 M citrate buffer (pH 4.5). The STZ-inoculated rats were allowed ad libitum 20% glucose solution for a duration of 24 h to avoid the occurrence of hypoglycemia. Rats of the control and NSO groups were injected with citrate buffer only [24]. The occurrence of diabetes was ascertained after 48 h post-STZ injection by determination of blood glucose level. The animals that possessed blood glucose values of over 250 mg/dL were supposed to be diabetic [25].

### 2.4. Sampling

After 30 days of treatments, overnight fasted rats were decapitated under effect of anesthesia, and blood samples were obtained in sterile plain tubes. The sera were stored at −20°C. Pancreas and liver of each experimental animal were excised, rinsed in cold phosphate buffered saline, and dried with filter paper. Pancreas and liver of each rat was divided into 2 parts: one part was kept at −80°C until preparation of pancreatic homogenate for reduced glutathione (GSH) and catalase assay. The remaining part of pancreas and liver was immersed in 10% neutral buffered formalin for histopathological and immunohistochemical examination.

### 2.5. Body Weight

Experimental rats were weighed weekly during the experimental period.

### 2.6. Blood Glucose Value and Insulin Level

Fasting blood glucose level was estimated using reagent strips (Accu-Chek®, Roche). The levels of insulin in serum were estimated by commercial rat enzyme-linked immunosorbent assay (ELISA) kit (Abnova, Germany) according to enclosed manufacturer's protocol.

### 2.7. Lipid Profile

The high-density lipoprotein cholesterol (HDL-c), low-density lipoprotein cholesterol (LDL-c), total cholesterol (TC), and triglycerides (TG) were measured in sera using enzymatic colorimetric kits (Stanbio Laboratory, USA, and ELITechGroup, France) according to Tietz [26].

### 2.8. Catalase and Reduced Glutathione Activity (GSH)

Catalase and GSH activities in pancreatic and hepatic homogenates were determined using colorimetric kits from Biodiagnostic (Egypt) and BioVision (USA), respectively, according to methods of Aebi [27] and Tietez [28], respectively.

### 2.9. Histopathology

Formalin-fixed pancreases and livers were put in paraffin wax, and several 5 *μ*m sections were sliced and then subjected to hematoxylin and eosin (H&E) stain according to Bancroft and Gamble [29]. Livers of 5 *μ*m sections were subjected to periodic acid Schiff (PAS) stain for glycogen demonstration [30].

### 2.10. Immunohistochemistry

Paraffin-embedded pancreases were sliced into 4 *μ*m sections on the positively charged slides. Sections were subjected to xylene deparaffinization and then rehydrated with descending series of ethanol, followed by water. The sections were incubated with primary monoclonal anti-insulin antibody (catalog no. 13-9769-80, Thermo Fisher Scientific, Canada) at a rate of 0.5 *μ*g/mL for 2 h at 25°C in a humidified chamber. Then the steps according to the method of Adewole and Ojewole [31] were followed.

### 2.11. Image Analysis and Islet Size

Measurements of pancreatic islet size were performed via ImageJ program. Insulin-positive islet immunostaining intensity and area % were determined from each slide of each experimental group using ImageJ program after subtracting light background. Seven fields of pancreatic islets were randomly chosen. The intensity of immunohistochemistry (IHC) staining and the percentages of IHC stained regions were obtained by ImageJ program and calculated according to Elgawish et al. [32]. The diameters of the pancreatic islet were measured by selecting seven of pancreatic islets/animal in all groups using ImageJ program by the aid of a calibrated micrometer.

### 2.12. Statistical Analysis

The data were presented as the mean ± standard error of mean (SEM). Statistically significant differences between groups were calculated using one-way analysis of variance (ANOVA) followed by Duncan's post hoc multiple comparison test (SPSS software, version 16.0; SPSS Inc., Chicago, IL, USA). The criterion for significance was set at *P* < 0.05.

## 3. Results

### 3.1. Body Weight

The body weights of the STZ-induced diabetic group rats reduced significantly (*P* < 0.05) than did those of the control nondiabetic rats at the 3rd and 4th weeks of the experiment. However, NSO 2 mL/kg treatment of diabetic rats increased their body weights (*P* < 0.05) compared to those of diabetic nontreated rats at the 4th week of the experimental period (Table 1).

### 3.2. Fasting Blood Glucose Value and Insulin Level

The injection of experimental rats with single i.p. dose of STZ induced a significant (*P* < 0.05) elevation in FBG levels compared to those of the control rats. Administration of NSO 2 mL/kg to the diabetic rats, for one month, induced a significant (*P* < 0.05) reduction in the levels of FBG compared to those of the diabetic group. Insulin level revealed a significant (*P* < 0.05) decrement in the STZ diabetic group than in the control one. Meanwhile treatment of the diabetic rats with NSO 2 mL/kg, for 1 month, significantly (*P* < 0.05) improved insulin to a level comparable to that of the control group (Table 2).

### 3.3. Lipid Profile

Oral administration of NSO 2 mL/kg to the nondiabetic rats for one month induced a significant (*P* < 0.05) increment in HDL-c level compared to that of the control group. However, HDL-c level revealed a significant (*P* < 0.05) decrement in the diabetic group than in the control. Meanwhile, administration of 2 mL/kg NSO to the diabetic rats increased HDL-c significantly (*P* < 0.05) compared to that of diabetic nontreated rats. Treatment of the control rats with NSO 2 mL/kg for one month induced a significant (*P* < 0.05) decrement in TC and LDL-c levels compared to those in the control. The diabetic rats revealed elevation in TC, TG, and LDL-c (*P* < 0.05) than did the control group. However, 30 days' treatment with NSO 2 mL/kg of the diabetic rats induced a significant (*P* < 0.05) decrement in TC, TG, and LDL-c values compared to those of the diabetic group (Table 2).

### 3.4. Pancreatic and Hepatic Oxidative Stress

Current data revealed that both hepatic and pancreatic catalase as well as GSH activities were decreased significantly (*P* < 0.05) in the STZ-induced diabetic rats than in the control. Administration of NSO 2 mL/kg, for one month, to the diabetic rats significantly (*P* < 0.05) elevated hepatic and pancreatic catalase and GSH activities compared to those in the diabetic group (Table 3).

### 3.5. Histopathology and Islet Size

**Figure 1:**
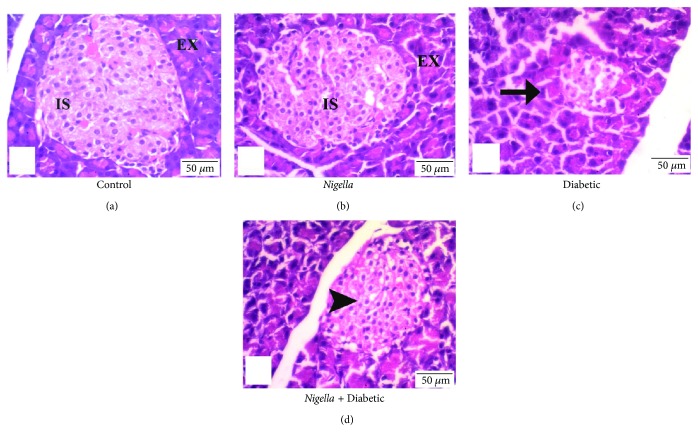
*Nigella* seed oil extract effect on pancreatic histopathology in normal and STZ-induced diabetic rats. (a and b) Control- and NSO- (2 mL/kg) treated control groups: normal control rat displayed healthy islets of Langerhans. (c) Diabetic control: arrow displays the atrophied islets of Langerhans. (d) NSO- (2 mL/kg) treated diabetic rats display bigger islets of Langerhans than do diabetic untreated group (arrowhead). Scale bar represents 50 *μ*m.

Examination of pancreatic sections from the control group and the *Nigella*-treated group showed abundant and scattered pancreatic islets of Langerhans between the exocrine parenchyma throughout the pancreas. The islets were large, well defined, and composed of groups of compact cells arranged in branching, irregular, and anastomosing cords separated by blood capillaries (Figures 1(a) and 1(b)).

On the other hand, examination of pancreases of the diabetic rats yielded an observable decrement in the number of islets. They were shrunken and irregular, and some of the islet cells showed hydropic degeneration while others were necrotized with deeply stained pyknotic nuclei. Few lymphocytes were seen attacking the islet cells (Figure 1(c)).

The diabetic group treated with oral NSO 2 mL/kg showed marked increase in number of islets throughout the pancreas, and most of them demonstrated recovery of their normal morphologic features comparable to those of the islets of the control group (Figure 1(d)).

Analysis of the mean diameter of pancreatic Langerhans islets among the studied groups showed a significant (*P* < 0.05) reduction in the mean diameter in the diabetic rats. Oral treatment with NSO (2 mL/kg), for one month, significantly (*P* < 0.05) increased the mean diameter of Langerhans islets compared to that in the diabetic nontreated group (Table 4).

**Figure 2:**
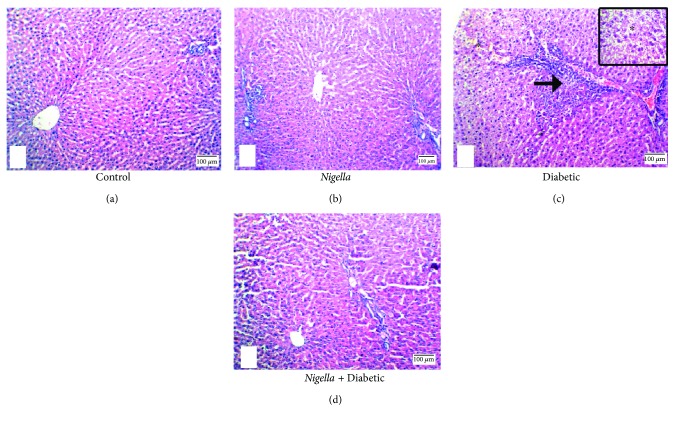
H&E stain of liver: (a) control group and (b) *Nigella*-treated rats demonstrate normal hepatic architecture showing the classic lobule with the central vein at the center and portal tracts at the periphery; each one is separated from the others by a similar distance, 100x. (c) Diabetic group showing ballooning degeneration of the hepatocyte (^∗^, 40x), and interface hepatitis (arrow), 100x. (d) NSO-treated diabetic group showing restoration of normal architecture, 100x. Scale bar represents 100 *μ*m.

The results obtained from histological sections of livers with hematoxylin and eosin staining for the control and *Nigella*-treated rats were similar. Liver histological observations of the control and *Nigella*-treated rats showed normal hepatic architecture showing the classic lobule with the central vein as its center and portal tracts at the periphery. Embedded in the portal tract were portal venule, hepatic arteriole, and interlobular bile ductule (Figures 2(a) and 2(b)). The livers of the diabetic animals showed ballooning degeneration, mild portal inflammation, and interface hepatitis (Figure 2(c)). Sections of liver tissues from diabetic animals treated with *Nigella* showed restoration of the normal hepatic architecture with marked reduction of portal inflammation and absence of interface hepatitis (Figure 2(d)).

**Figure 3:**
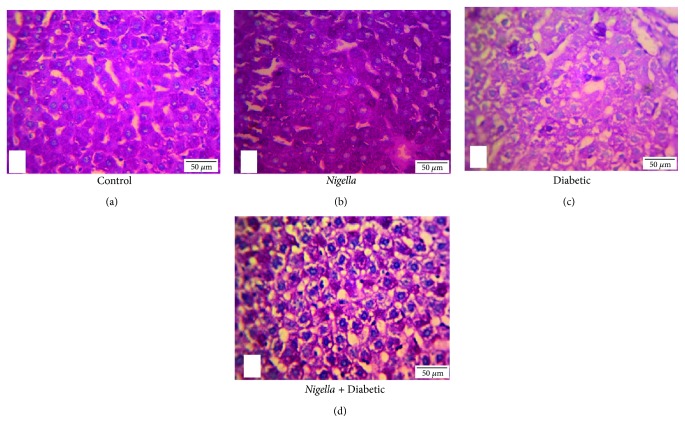
PAS stain of liver: (a) control group and (b) NSO- (2 mL/kg) treated control rats demonstrate abundant glycogen, which is present within the cytoplasm of the hepatocytes (magenta-colour granules), 400x. (c) Diabetic group demonstrates marked reduction in PAS positivity (few scattered glycogen granules), 400x. (d) NSO- (2 mL/kg) treated diabetic group demonstrates restoration of PAS positivity, 400x. Scale bar represents 50 *μ*m.

The PAS-stained liver sections demonstrated abundant cytoplasmic contents of glycogen in the control and NSO-treated control (Figure 3(a) and (b)). The diabetic group showed marked depletion of glycogen contents in hepatocytes cytoplasm than did the control ones (Figure 3(c)). Treatment of diabetic rats with 2 mL/kg NSO improved glycogen contents in hepatocytes cytoplasm compared to those in the diabetic rats (Figure 3(d)).

### 3.6. Immunohistochemistry

Positive insulin expression was visualized as dark-brown cytoplasmic granules in the pancreatic islets' *β* cells. The quantitative analysis of insulin immunoreaction in the pancreatic islets showed significant (*P* < 0.05) variations among the study groups. Regarding the percentage of insulin-positive islet area, the analysis showed a significant (*P* < 0.05) decrement in the percentage of insulin-positive area in the diabetic group than in the control group. However, NSO treatment of the diabetic rats significantly (*P* < 0.05) improved positive islet area % compared to that of the diabetic ones (Table 4).

**Figure 4:**
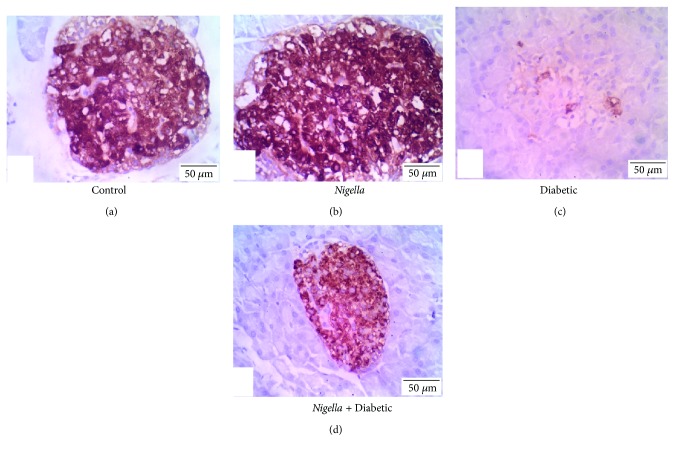
Immunohistochemical staining for insulin expression in pancreatic islets from different experimental groups. (a) Control, (b) NSO- (2 mL/kg) treated group, (c) diabetic group, and (d) diabetic group treated with NSO (2 mL/kg). Scale bar represents 50 *μ*m.

The immunostaining intensity of pancreatic islets' insulin was assessed in a quantitative fashion according to the microdensitometric method. The staining intensities of insulin in the pancreatic islets among different study groups are shown in Figure 4 and Table 4. The *Nigella*-treated group exhibited pancreatic islets with the highest insulin staining intensity followed by the control with no significance between both of them compared to the diabetic group treated with NSO. On the other hand, the diabetic untreated animals showed the lowest (*P* < 0.05) insulin staining intensity.

## 4. Discussion

Diabetes mellitus is considered as one of the systemic, endocrine, and metabolic diseases, which is diagnosed by hyperglycemia. This could be considered with alterations in carbohydrate, lipid, and protein metabolism. This is considered to be due to reduction and/or impairment in antioxidant mechanisms inside the body leading to weight loss [33, 34].

Therefore the usage of antioxidants is considered as one of the strategies used for resetting the normal homeostasis in diabetic patients [35, 36]. Numerous plant extracts and compounds from these extracts are known to possess antidiabetic properties that could be used in the remedy of diabetes mellitus [11, 37]. *Nigella sativa* thymoquinone is considered as an antioxidant and hypoglycemic compound that could counteract the high cost and the adverse effects of pharmacological drugs [38], which confirms the antidiabetic effects of NSO.

The current study demonstrated that STZ produced a significant (*P* < 0.05) decrement in body weights at the 3rd and 4th weeks of experiment. These results are considered to be consistent with Wong and Tzeng [39] and Doğan and Çelik [40]. This could be due to reduction in glucose availability as well as amino acids to the body cells, which creates a lack in the substrates that are necessary for cellular biosynthesis and can affect linked cellular metabolism that causes muscle wasting [39]. Oral administration of NSO (2 mL/kg) containing 5% thymoquinone to the diabetic rats led to a marked increment in the body weights of these animals compared to diabetic nontreated rats. The current results were in accordance with those of Kanter et al. [41] and Kaleem et al. [42]. This may confirm the effect of NSO on diabetes-induced muscle wasting due to carbohydrate inaccessibility, which confirms the antidiabetic effect of NSO [42].

The serum level of insulin was reduced in the diabetic rats than in the control, while FBG level was increased (*P* < 0.05) in the diabetic group than in the control one. These results are considered to be consistent with those of Houcher et al. [43] and Mahmoud et al. [24]. This effect is due to the alkylating toxic action of STZ on *β* cells of pancreatic islets, which blocks insulin secretion leading to hyperglycemia [44]. NSO thymoquinone induced a marked decrease and a marked increase at *P* < 0.05 in both FBG and insulin levels, respectively, than in those in the diabetic group. These results were consistent with those of Fararh et al. [45], Rchid et al. [46], Mahmoud et al. [24], and Balbaa et al. [25]. The FBG-lowering effect of NSO thymoquinone may illustrate its insulinotropic action [47] where it can make partial regeneration to *β* cells of pancreatic islets, thus leading to improvement in their insulin production [48] as well as its peripheral utilization [49]. Thymoquinone has a downregulating effect on the gluconeogenic enzymes expression and the production of hepatic glucose besides its ability for diminishing intestinal absorption for glucose [23]. Moreover, it can activate adenosine monophosphate-activated protein kinase (AMPK) in muscles and liver, thus inhibiting gluconeogenesis [50].

As clarified in the current study, STZ-induced dyslipidemia in the diabetic group (decreased HDL-c and increased TC, TG, and LDL-c). These results were parallel to those obtained by Doğan and Çelik [40]. Diabetic dyslipidemia occurs as a consequence to insulin deficiency and hyperglycemia, which increases lipolysis and fatty acids release from adipose tissue into circulation with alteration in their metabolism [51], thus increasing TC, TG, and LDL-c levels that predispose cardiovascular pathology [52]. Administration of NSO has positive effects on diabetes-induced abnormal lipid profile, which coincides with the results of Kaleem et al. [42], Kocyigit et al. [53], and Balbaa et al. [25]. The ameliorating effects of NSO thymoquinone on diabetic dyslipidemia may be due to its promoting effect on hepatic arylesterase activity, regulatory effects on cholesterol metabolism-influencing genes (Apo-A1, Apo-B100, and LDL-receptor genes) [54], and its antioxidant properties [55, 56].

In the current study, the influence of NSO on pancreatic and hepatic oxidative stress was examined in the STZ-induced diabetic male rats. The underlying mechanisms behind the oxidative stress and the free radicals generated by diabetes have been extensively investigated [41, 57]. The diabetic rats exhibited significantly reduced pancreatic and hepatic catalase and GSH activities due to the recorded hyperglycemia and hyperlipidemia [58], which exhaust the activities of natural antioxidants and promotes free radicals formation [59]. Treatment with NSO thymoquinone improved the antioxidant reserve of GSH and catalase activities in pancreatic and hepatic tissues, which was in agreement with the findings of Meral et al. [60], Kanter et al. [41], and Adewole et al. [61]. The antioxidant potential of thymoquinone molecule is confirmed by the presence of quinine in its structure [62]. Quinine facilitates the efficient access to the cellular and subcellular structures, making the ROS elimination easier [63]. Thymoquinone can also inhibit nonenzymatic lipid peroxidation [54] that protects hepatic and pancreatic antioxidant enzymes and reduces oxidative stress [15]. Moreover, the hypoglycemic influence of NSO potentiates its antioxidant effect via modulating hyperglycemia-induced ROS.

Parallel to the results of hepatic and pancreatic antioxidant enzymes, the hepatic and pancreatic histopathology showed deleterious effects on the STZ-induced diabetic rats. These changes include hepatic congestion, fibrosis, centrilobular hepatocyte swelling, and incidence of inflammatory cell infiltration, which were ameliorated by NSO. Pancreatic pathology revealed severe atrophy, necrosis of acinar cells, and severe reduction in islet area %, which were greatly improved by NSO administration. The present results were in accordance with those of Kanter et al. [41], Widodo et al. [64], and Tuhin et al. [65]. The improvement in both pancreatic and hepatic histopathology could be attributed to the antioxidant potential of thymoquinone in NSO. Thymoquinone improves diabetic depletion of antioxidant reserves of catalase and GSH, thus keeping the integrity of both hepatic and pancreatic cells.

The depletion of hepatic glycogen contents in the diabetic rats was due to depletion of insulin that led to increased gluconeogenesis rather than glycogenesis [66]. Administration of NSO 2 mL/kg to the diabetic rats markedly improved hepatic glycogen contents due to the promotion of pancreatic insulin secretion, which activates glycogen synthase enzyme, thus reducing circulating glucose level [67].

The increment in pancreatic islet immunostained area % by NSO treatment in the diabetic rats, as a result of its antioxidant effect, was in agreement with the findings of Omar and Atia [38] and Widodo et al. [64]. The improvement of insulin immunoreactivity as well as pancreatic islet morphology and diameter could elucidate the upregulation of serum insulin level and the decrease in FBG, thus improving hyperlipidemia in diabetic rats.

## 5. Conclusion

Based on the current results, NSO was found to be an effective protection against the adverse consequences of STZ-induced diabetes in Wistar rats. NSO exerts its effect through thymoquinone antioxidant potential, which improved pancreatic and hepatic integrity, thus increasing pancreatic islet immunoreactivity, therefore increasing the serum insulin level, increasing hepatic glycogen content, reducing the elevated blood glucose level, and counteracting diabetic dyslipidemia.

## Figures and Tables

**Table 1:** The effect of NSO 2 mL/kg on weekly body weight (g) of STZ-induced diabetic rats.

	Groups	Control	*Nigella*	Diabetic	Diabetic *Nigella* treated
Body weight (g)	Week 1	200.50 ± 1.48^a^	200.33 ± 1.60^a^	201.17 ± 1.72^a^	201.50 ± 2.08^a^
	Week 2	212.50 ± 3.23^a^	211.25 ± 2.46^a^	205.50 ± 2.10^a^	203.25 ± 4.59^a^
	Week 3	220.00 ± 4.56^a^	228.75 ± 4.92^a^	195.00 ± 3.19^b^	200.75 ± 4.91^b^
	Week 4	226.75 ± 5.27^a^	239.00 ± 3.56^a^	160.00 ± 4.^c^	188.50 ± 3.12^b^

Values are presented as mean ± SE. ^abc^Different letters on numbers in each row represent a significant difference at *P* < 0.05.

**Table 2:** Effect of NSO 2 mL/kg on FBG (mg/dL), insulin (pmol/L), HDL-c (mg/dL), LDL-c (mg/dL), TG (mg/dL), and TC (mg/dL) in STZ-induced diabetic rats.

Groups	Control	*Nigella*	Diabetic	Diabetic *Nigella* treated
FBG (mg/dL)	98.40 ± 7.00^c^	93.60 ± 3.80^c^	313.00 ± 5.19^b^	187.40 ± 19.91^a^
Insulin (pmol/L)	5.78 ± 0.49^a^	6.25 ± 0.47^a^	3.41 ± 0.29^b^	5.36 ± 0.27^a^
HDL-c (mg/dL)	44.73 ± 1.82^b^	53.37 ± 2.02^a^	22.31 ± 2.45^d^	33.60 ± 2.26^c^
LDL-c (mg/dL)	56.91 ± 2.86^c^	47.09 ± 2.60^d^	109.28 ± 4.18^a^	84.52 ± 2.99^b^
TG (mg/dL)	58.28 ± 3.47^c^	52.07 ± 4.14^c^	105.92 ± 3.19^a^	84.25 ± 2.81^b^
TC (mg/dL)	109.96 ± 4.08^c^	89.84 ± 4.77^d^	161.28 ± 8.54^a^	139.54 ± 3.92^b^

Values are presented as mean ± SE. ^abcd^Different letters on numbers in each row represent a significant difference at *P* < 0.05.

**Table 3:** Effect of NSO 2 mL/kg on catalase (U/g) and GSH (mg/g) in pancreas and liver of STZ-induced diabetic rats.

Groups	Control	*Nigella*	Diabetic	Diabetic *Nigella* treated
Pancreatic catalase (U/g)	84.27 ± 5.56^a^	86.66 ± 4.91^a^	38.25 ± 2.30^c^	69.00 ± 1.22^b^
Hepatic catalase (U/g)	27.49 ± 2.26^b^	34.73 ± 2.09 ^a^	10.51 ± 1.31^d^	18.19 ± 1.09^c^
Pancreatic GSH (mg/g)	36.45 ± 2.80^a^	42.71 ± 4.49^a^	13.49 ± 3.12^c^	23.00 ± 0.95^b^
Hepatic GSH (mg/g)	85.71 ± 9.57^a^	84.92 ± 6.33^a^	35.95 ± 6.32^b^	62.89 ± 6.63^a^

Values are presented as mean ± SE. ^abcd^Different letters on numbers in each row represent a significant difference at *P* < 0.05.

**Table 4:** The effect of NSO 2 mL/kg on mean pancreatic islet size (*μ*m), insulin-positive islet area %, and insulin immunostaining intensity (integrated density) in STZ-induced diabetic rats.

Groups	Control	*Nigella*	Diabetic	Diabetic *Nigella* treated
Mean islet size (*μ*m)	233.90 ± 24.71^a^	247.52 ± 35.27^a^	29.83.36 ± 83.17^b^	170.61 ± 26.63^a^
Insulin-positive islet area (%)	65.79 ± 9.81^a^	68.52 ± 9.58^a^	18.01 ± 3.42^c^	43.05 ± 5.57^b^
Insulin immunostaining intensity (integrated density)	92.50 ± 3.05^a^	99.34 ± 2.23^a^	60.93 ± 4.31^c^	76.15 ± 1.92^b^

Values are presented as mean ± SE. ^abc^Different letters on numbers in each row represent a significant difference at *P* < 0.05.
